# Does the Economic Growth Improve Public Health? A Cross-Regional Heterogeneous Study in China

**DOI:** 10.3389/fpubh.2021.704155

**Published:** 2021-06-18

**Authors:** Xiao-Tong Niu, You-Cai Yang, Yu-Cong Wang

**Affiliations:** School of Economics and Management, Qingdao University of Science and Technology, Qingdao, China

**Keywords:** economic growth, heterogeneous, panel threshold regression, public health, China

## Abstract

On public health, the effect of economic growth in China is analyzed in this paper by using the panel threshold regression model. The empirical study from 2000 to 2017 shows that China's economic growth has a significant threshold effect on public health. After the threshold is exceeded, public health will be improved dramatically. The threshold effect is heterogeneous at the regional level. The eastern region has no threshold, and both central and western regions have a single significant threshold. However, the threshold value and threshold effect in the central and western regions are also different. The heterogeneity is caused by the different levels of regional economic development. Therefore, based on public health utility maximization, the government should make different health policies according to the characteristics of regional development.

## Introduction

This study aims to see if economic growth (defined as GDP per capita, lnpgdp) is affected by public health (defined as health expenditure, PH) and if regional heterogeneity exists. People who are in good health are more efficient economically. Since healthy people expect to live longer and are naturally more worried about their potential financial needs, better health leads to higher savings rates. Education is another connection between health and the economy. With the economy's growth, various medical policies have been constantly implemented, and people's living standards have improved, which causes public health to become a topic of concern (1–3). China, the world's second-largest economy, with its rapid economic growth, promotes the continuous improvement of national health policy and healthcare system, as well as a substantial increase in health spending. Since the medical reform started in 2009, China has achieved nearly universal health coverage for 95% of the population, more than 1.3 billion people (4). In 2016, the New Rural Cooperative Medical Scheme and the Basic Medical Insurance for Urban Residents were merged into the Basic Medical Insurance for Urban and Rural Residents. The merger reduced the inequality in reimbursement and improved the service efficiency in China (5). Economic growth promotes the increase in health expenditures, which can be explained in the following two ways. First, as the country's economy grows, the scale of public health expenditures and financial subsidies brought about by the improvement of the medical system will increase year by year (6, 7). Second, economic growth has greatly increased residents' incomes. Life quality gets guaranteed, people pay more attention to health issues, and health-related expenditures are increased (8–10). The improvement of public health will promote the improvement of people's work efficiency and the extension of working hours, which will promote economic growth and form a virtuous circle (11–13).

Can economic growth improve public health? Preston found that as early as 1975, economic development level is one of the important factors affecting public health level. Although there is no unanimous conclusion, most researchers believe that it positively relates to public health. Gerdtham and Jönsson (14) reveal that GDP per capita contributes significantly to explaining the healthcare expenditure's variation. Braendle and Colombier (15), Gürler and Özsoy (16), and Shahbaz et al. (17) demonstrated this. Other scholars believe that economic growth fails to improve public health and inhibits the improvement of public health. Alves et al. (18) and Pope et al. (19) argued that economic growth had been accompanied by industrialization and urbanization. With the worsening of environmental pollution, people's health is threatened. Acemoglu and Johnson (20) and Ruhm (21) believed that the rising unemployment rate could effectively reduce the death rate. The threshold model is used in this study to examine the impact of economic growth on the public's health. Is there any regional heterogeneity in how economic growth affects public health? In areas with a high level of economic development, welfare policies are higher, and the public health policies and healthcare systems provided are more complete (22–24). Popham et al. (25) argued that Scandinavia's high economic level enables it to be the most developed welfare state, and its public health advanced worldwide. Robinson et al. (23), Rydland et al. (24), and Thompson (26) also believed that different from areas with high economic growth, low economic development regions have poor health security coverage and limited access to health services, resulting in poor public health (27, 28). Babitsch et al. (29) believed that health expenditure is lower in lower socioeconomic groups, even though their health needs are higher. Therefore, the impact of economic growth on public health is heterogeneous. Health impacts are different between developed and developing countries (30).

**Figure 1:**
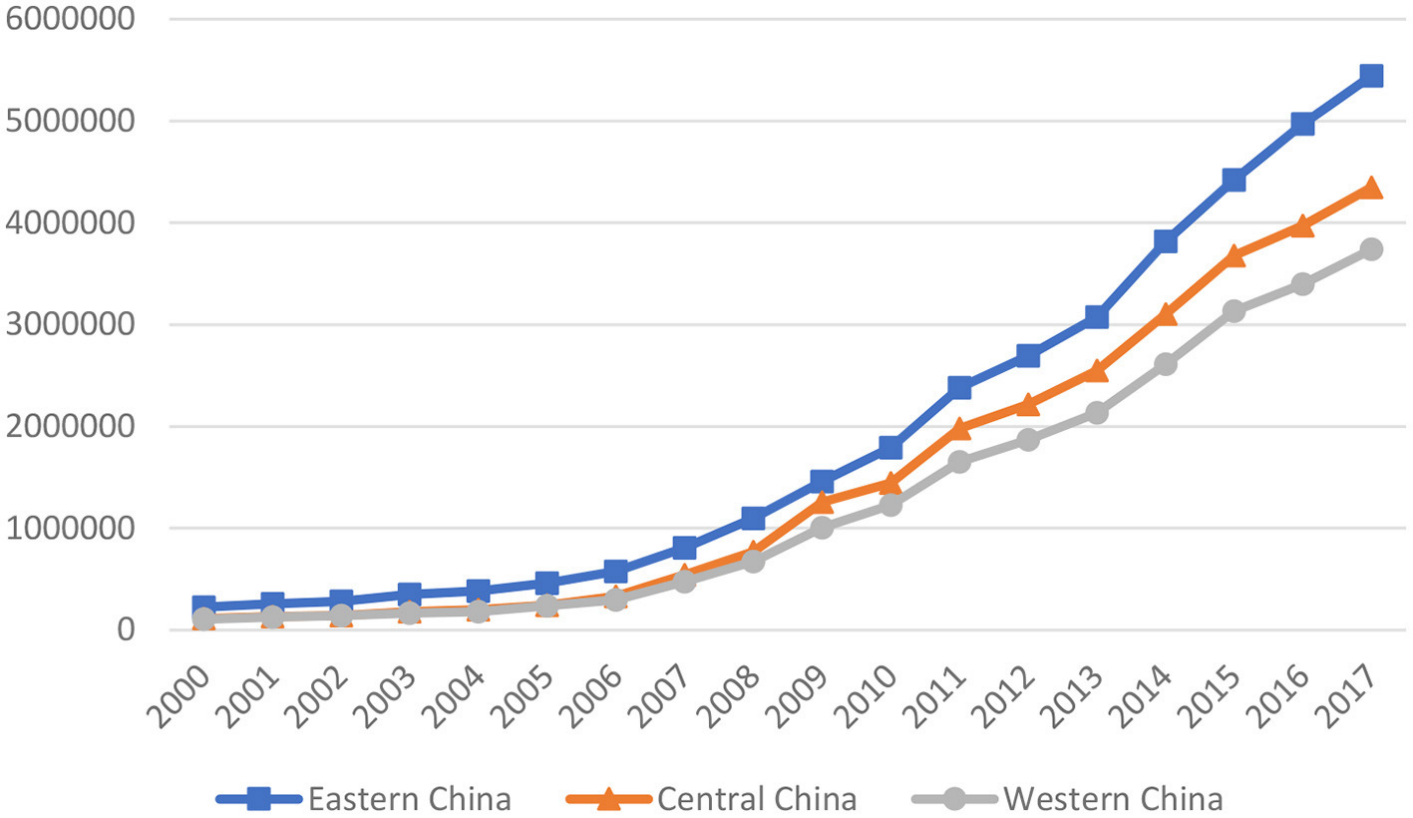
Average medical and health expenditures in eastern, central, and western China from 2000 to 2017.

China's economy develops rapidly, where science and technology have also made great progress. At the same time, people's living standards and nutritional status have greatly improved. Theoretically speaking, China's medical and health services have great development. The government has an inescapable responsibility for the medical and health market, subsidizing and intervening in the medical and health market through its financial activities or administrative management methods. The number of health technicians per 1,000 people in China rises from 3.63 in 2000 to 7.26 in 2019, and the growth rate is 134.05%. The COVID-19 pandemic has precipitated a global crisis due to the continued absence of a vaccine or cure. With the economic growth and technological progress, China makes extensive use of mobile health technology to collect medical data and provide health services, which is critical in combating the COVID-19 epidemic (31). This study examines the relationship between economic growth and public health in China, the world's most populous developing country with a population of more than 1.3 billion people, using literature to help policymakers understand better in developing countries the economic growth effect on public health and develop targeted policies. China is a vast country, and different regions have great differences in economy, society, ideology, and geography. The 30 provinces of China can be divided into three regions—eastern China is highly developed, central China is moderately developed, and western China is least developed (22). Different levels of economic development led to differences in medical policies and health expenditure among regions, which further leads to regional differences in health conditions (27). The eastern region has the best medical and healthcare services, while the central and western regions have relatively balanced medical resources. In 2000, there were 2.084, 1.689, and 1.685 health technicians per 1,000 residents in the eastern, central, and western areas, respectively. This indicator had risen to 2.68, 2.37, and 2.33 by 2017. As a result, the effect of economic growth on public health varies significantly across regions. Therefore, it is necessary to divide China into three major categories, east, central, and west, and conduct heterogeneity analysis to make the research conclusions more accurate (See Figure 1).

Can economic growth always promote public health? Most studies have given relevant favorable evidence. Even so, is this promotion linear? Does the promotion effect increase or decrease with the difference of economic growth level? Is there, in other words, a threshold effect? Is its promotion impact heterogeneous even across different regions of the same country? As a consequence, this paper makes the following contributions to the study of the above problems. To begin with, there is a limit to China's economic growth and public health. When the threshold is reached, economic growth has the potential to boost public health dramatically. Secondly, from a regional perspective, the impact of economic growth on public health is linear in the eastern region. There is no threshold effect, but it appears as a threshold effect in the central and western regions. This may be due to the high level of economic development in the eastern region leading to good medical conditions, which better meet the people's requirements for public health. On the contrary, the economic development of the central and western regions is underdeveloped, the government's financial support for public health is limited, poor medical infrastructure and other reasons caused the region's economic growth to exceed the threshold, and its promotion of public health has increased significantly. Third, although the central and west have a threshold, the threshold is different, and the promotion is also different. The specific manifestation is that the threshold for central China is higher than that in the west.

The remainder of the analysis is organized as follows: section Literature Review shows the review of related papers. The relationship between economic growth and public health is revealed in section Economic growth with health utility model. The empirical methods are discussed in section Methodology. The data is presented in section Data. The empirical analysis is presented in section Empirical Results. This paper's research is summarized in section Conclusions.

## Literature Review

On the effect of economic growth on public health, there are two points of view. Economic growth, it is widely assumed, enables people to live better, longer lives and enjoy good health. Most studies agree that economic growth boosts public health. Health expenditure is often used as a measure of public health. Hamoudi and Sachs (32) believed that economic growth increases health expenditure. Endrei et al. (33) confirmed that the economic environment strongly influences health expenditures in Hungary. Following the outbreak of world economic crises, the government health insurance expenditures decreased significantly. Braendle and Colombier (15) showed a positive correlation between per capita income and growth in healthcare spending. Gerdtham and Jönsson (14) pointed out that among the 19 OECD countries, per capita GDP levels have different effects on health expenditures. In developing countries, there is a strong association between economic growth and health spending. Hone et al. (8) studied that economic crisis in Brazil causes a negative effect on healthcare access. Hone et al. (8) pointed out that delays in pay medical staff, medicine shortages, and clinic closures cause reduced health expenditure. Mortality is also used as a measure of public health. “Preston curve” means that economic growth and population life expectancy have a strong correlation. The life span of people in areas with high economic development is longer than that in areas with low economic development. Coope et al. (34) suggested that economic strain may increase the suicide rate of men aged 35–44. Haw et al. (35) explained this phenomenon because the effect of recession causes unemployment, job insecurity, financial loss, bankruptcy, and home repossession. According to Erdogan et al. (36), there is an important and negative association between infant mortality and real per capita GDP in high-income OECD countries.

Many scholars have begun to study the mechanism by which economic growth positively impacts public health. First, economic growth can promote the improvement of the local welfare system, thereby improving public health. Welfare regimes are an established macro determinant for public health (27, 37). Kautto et al. (38) explained that welfare policies had reduced inequalities in income, housing quality, healthcare access, and other social and economic outcomes. Eikemo et al. (28) also found that population health varies significantly by welfare state regime. Bergqvist et al. (39) claimed that flexible policies and incentives positively impact everybody in a population's well-being.

Similarly, Hall and Lamont (40) claimed that public policy improves health through economic redistribution and strengthening individuals' and communities' social resources. Youkta and Paramanik (41) suggested that the government's extent and pattern are politically driven. Political factors play a significant role in determining health expenditure. Second, economic growth has the potential to increase people's income levels; there is no question that increased income levels would boost public health (42). According to Fogel (43), economic growth contributes to higher income and a higher quality of life, which improves health. Summers and Pritchett (44) believed that an increase in per capita income could reduce infant deaths. Hamoudi and Sachs (45) found that in developing countries, even AIDS-endemic countries, higher income levels lead to significant improvements in public health. Third, a broad consensus holds that economic growth pushes forward technological progress, including medical technology advancement, from which results in health levels are improved (9). Dreger and Reimers (46) analyzed accounts that health expenditures are not driven solely by income but also by medical progress. According to Fuchs (47), the rapid growth in healthcare spending in the United States over the last three decades has been propelled by technological change, as indicated by Newhouse (48). Newhouse (48) also pointed out that technological advancement is a major factor in healthcare costs. According to Newhouse (48), technological progress is the driving force behind healthcare spending in various countries. Another view is that economic growth inhibits public health. Many studies are showing that economic growth also increases environmental pollution, endangering public health. Scholars pay close attention to the impact of environmental pollution on public health (49–51). Deryugina et al. (52) supported that severe environmental pollution is a major risk factor that affects public health. Alberini et al. (53) demonstrated that more pollution in the city would cause higher mortality. In the context of China, Fan et al. (54) estimated that the air quality index is significantly related to mortality. According to Gehring et al. (55), reduced levels of air pollution can help prevent the development of asthma in children.

As one can see, most scholars believe that economic growth can significantly promote the level of public health, but some scholars believe that economic growth leads to serious pollution and environmental damage and damages the level of public health. Economic growth on health is different in different countries and regions, and it is challenging to form consistent empirical conclusions and systematic theoretical explanations. This article can enrich relevant research. In addition, most studies take a single country as the research object and analyze the impact of a country's overall economic growth on public health, failing to consider regional heterogeneity. This article takes China as the research object and divides it into three regions. The specific analysis of the regional economic development stage and other basic development issues obtains conclusions and opinions with a reference value and proposes more feasible ways to improve public health—level of policy recommendations. Finally, most of the studies between the two have chosen quadratic functions. This article uses the threshold model to study the relationship between the two and make the research conclusions more accurate.

## Economic Growth With Health Utilityodel

Using Ramsey (56) and Tai et al. (57) model, we have described the effect of economic growth on public health. Many companies are the same. To produce and sell output, competitive firms rent capital and hire workers, and each has exposure to the production function Y = F (K, AL), which meets Inada conditions. There are four variables, output (Y), capital (K), labor (L), and knowledge (A), where the growth rate of A is g and that of L is zero. So, the marginal capital product (γ(t)) is ∂F(K,AL)∂K and the marginal product of labor ∂F(K,AL)∂L. A fixed amount of infinitely lived households includes labor supply, capital holding, consumption, and savings. The form of the household's utility function is changed to $\text{U}=\int_{t=0}^{\infty }e^{-\rho t}u(H,C)dt$, where H is the health consumption and C is the general consumer, which means that the happiness of consumers depends not only on the consumption of general goods but also on the expenditure on health. *u*(*H, C*) is the instantaneous utility function, and ρ is the discount $u\left(H,C\right)=\frac{{\left(H+C\right)}^{1-\eta }}{1-\eta },\eta <0,\rho -\left(1-\eta \right)g>0$.

The evolution of the capital is

(1) $$\overset{•}{\text{K}}=Y-C-H-\delta \text{K}$$

where capital depreciates at a rate δ.

The objective of the household can be expressed by the following:

(2) $$\begin{array}{l} Max\int_{t=0}^{\infty }e^{-\rho t}u(H,C)dt\;\text{subject to}\;\int_{t=0}^{\infty }\text{-R(t)}(\text{H}+C)dt\\ \;\leq Y(0)+\int_{t=0}^{\infty }e^{-\text{R}(\text{t})}W(t)dt\;\text{and }{\underset{s\to \infty }{\lim e}}^{-R(s)}K(s)\geq 0\; \end{array}$$

where $R(t)=\int_{\tau =0}^{t}\gamma (\tau )d\tau$ .

The first-order condition implies the Euler equation:

(3) $$\frac{\left(\dot{H}+C\right)}{H+C}=F(Y,g,\eta ),$$

Therefore, health spending is the function of GDP and technology. Suppose that H is proportional to C, then

$$\frac{\Delta H}{H}=F(Y,g,\eta ),$$

thus healthy consumption depends on economic growth and technological progress. Moreover, Ruhm tells us that health is the function of pollution (P), medical care (M), etc. It means $\frac{\Delta H}{H}=F\left(Y,g,\eta ,P,M\right)$. Therefore, economic growth will lead to the change of health expenditure, which may be linear or non-linear and may be the threshold effect of structural change.

## Methodology

Compared with the quadratic non-linear functional relationship, the non-linearity of the threshold model is determined by whether there is an endogenous threshold. The model is made up of three phases in general: (i) endogenous threshold values are estimated first, which avoids the need for an arbitrary classification scheme Hansen (58) and thus increases the validity of the results; (ii) thresholds (multiple or single) are used so that country samples can be segmented to classify (or “phases” in this model); and (iii) associations between outcome variables and the explanatory variables are finally established (phase).

The foundation for constructing more complex models is a one-threshold model. As a result, we examine the non-linear association between health spending and economic growth in this analysis using the Hansen (58) panel single-threshold regression model. An equation for the one-threshold model with {*PH*_*it*_, *LNpgdp*_*it*_, *x*_*it*_:1 ≤ *i* ≤ *n*, 1 ≤ *t* ≤ *T*} is

(4) $${\text{PH}}_{\text{it}}=\{\begin{array}{rr} \mu_{\text{i}}+\beta_{\text{1}}{\text{lnpgdp}}_{\text{it}}+\alpha_{1}^{\prime }{\text{x}}_{\text{it}}+\varepsilon_{\text{it}}, & \text{if }{\text{GDP}}_{\text{it}}\leq \gamma \\ \mu_{\text{i}}+\beta_{\text{2}}{\text{lnpgdp}}_{\text{it}}+\alpha_{2}^{\prime }{\text{x}}_{\text{it}}+\varepsilon_{\text{it}}, & \text{if }{\text{GDP}}_{\text{it}}>\gamma \end{array}$$

Where *PH*_*it*_ denotes health expenditure for country i in year t; *lnpgdp*_*it*_ is the threshold variable which is the logarithm of per capita real GDP; the estimated threshold value is γ; the threshold coefficients are β_1_ and β_2_; x_it is the control variable; and the control variable coefficients are α_1_ and α_2_; and the fixed effect in different countries under different conditions is denoted by μ_it_. ε_it_ is a white noise machine that conforms to ε_it_ ~ (0, σ^2^); the countries and time intervals are denoted by i and *t*.

The following formula can be written by modifying Equation (4):

(5) $$\begin{array}{l} {\text{PH}}_{\text{it}}=\mu_{\text{i}}+\beta_{\text{1}}{\text{lnpgdp}}_{\text{it}}\psi ({\text{lnpgdp}}_{\text{it}}\leq \gamma )\\ \text{ +}\beta_{\text{2}}{\text{lnpgdp}}_{\text{it}}\psi ({\text{lnpgdp}}_{\text{it}}>\gamma )+\alpha^{\prime }{\text{x}}_{\text{it}}+\varepsilon_{\text{it}} \end{array}$$

where ψ (.) are functions taking the value of 0 or 1.

In order to quantify the average health of each sample country, μ_i is removed using Eq. (5), resulting in:

(6) $$\begin{array}{l} {\text{PH}}_{\text{i}}=\mu_{\text{i}}+\beta_{\text{1}}{\text{lnpgdp}}_{\text{it}}\psi ({\text{lnpgdp}}_{\text{it}}\leq \gamma )\\ \text{ +}\beta_{\text{2}}{\text{lnpgdp}}_{\text{it}}\psi ({\text{lnpgdp}}_{\text{it}}>\gamma )+\alpha^{\prime }{\text{x}}_{\text{it}}+\varepsilon_{\text{it}} \end{array}$$

## Data

The empirical analysis in this study estimates in China the impact of economic growth on public health using panel data from 30 provinces from 2000 to 2017. China has been vigorously pursuing an opening strategy that has increased economic growth since the year 2000. China's accession to the World Trade Organization in 2001 made it become one of the major economies in the world (59). Therefore, the research in this paper started in 2000. The data sources are the China Statistical Yearbook, China Industry Statistical Yearbook, and China Health Statistics Yearbook. Our dataset contains the following annual macro-variables: lnpgdp as an economic growth indicator and a threshold variable (2, 26, 57), and health expenditure (PH) as public health (33, 42, 46).

Regarding public health, an evident and important stylized fact is the widespread rise in health spending. The medical and health expenditure in the sample data was 70.952 billion yuan in 2000 and rose to 1520.58 billion yuan in 2017, an increase of 204.3%. Specifically, the average values of the eastern, central, and western regions in 2000 are 2.25, 1.13, and 1.06 billion yuan, respectively. In 2017, the average values were 54.45, 43.49, and 37.398 billion yuan. All three regions showed an upward trend in the same proportion, with the highest in the east and the lowest in the west.

This study introduces six control variables. The first is mortality (ML); mortality refers to the ratio of the number of dead individuals in a certain period to the average population in the same period in a region. Mortality can judge the health habits and medical quality of a region, and it has a close relationship with public health. The disposable income per capita (CPI) is the second component. The CPI is an economic index representing the direction and magnitude of price shifts in consumer goods and services that affect people's lives (28). SO_2_ pollution (EP) harms vegetable growth and poses a severe threat to public health (1), resulting in significant social and economic losses in China. R&D spending (TP) is the fourth variable; technological advance is critical to public health (31). Low value added, high emissions, and energy intensity characterize the secondary industry, contributing to environmental degradation (53). Hence, the share of secondary industry in GDP and the total industrial output value is introduced as an explanatory variable in this study (20).

The descriptive statistics are shown in Table 1. To define the non-linear relationship and reduce the effects of heterogeneity, all variables are in logarithmic form. The mean values of the five indicators, including PH, lnpdp, TP, IO, and SI, are highest in the east, followed by the middle and the lowest in the west. Specifically, the logarithmic average values of medical and health expenditure in eastern, central, and western China are 13.79, 13.625, and 13.248, respectively. The logarithmic average per capita GDP of eastern, central, and western China is 10.358, 9.803, and 9.654, respectively. There is a significant divide between the eastern and western areas due to the highest level of economic growth among the three regions. The eastern region has the largest average R&D expenditure. During the sample period, the eastern region has a higher industrialization level and a faster development speed, followed by the central region and the western region. There is a big gap between the eastern and western regions. Among the other three variables, mortality in the western region is the highest at 6.186, that in the central region is 6.051, and that in the eastern region is 5.877. This is due to the economic development in the east and the high level of medical care (33). The average value of the consumer price index is about 102, indicating little difference between the eastern, central, and western regions. SO_2_ emissions are 4.242 in the central region, 3.946 in the west region, and 3.721 in the east region; this is due to the relevant national environmental policy which promoted the east area of environmental regulation, strengthened environmental protection, including the government subsidies, and improved technology of enterprises and other means to control the emissions of SO_2_ (19) effectively. Health spending is skewed to the left in every survey data structure. All of the data series have a normal distribution, according to the Jarque-Bera test findings.

**Table 1:** Descriptive statistics of the variables.

		**Obs**	**Mean**	**Std. dev**.	**Min**	**Max**	**Skewness**	**Curtis**	**Jarque-Bera**
**China**	PH	540	13.578	1.346	10.065	16.386	−0.225	2.131	21.575
	lnpgdp	540	9.980	0.810	7.922	11.832	−0.138	2.224	15.258
	ML	540	6.021	0.692	4.210	7.695	−0.399	2.766	15.599
	CPI	540	102.263	2.035	96.700	110.090	0.453	3.216	19.537
	EP	540	3.944	0.901	0.357	5.299	−1.366	5.284	28.488
	TP	540	13.582	1.635	9.024	16.969	−0.320	2.684	11.463
	IO	540	9.213	1.148	5.978	11.667	−0.260	2.696	8.180
	SI	540	8.066	1.194	4.393	10.574	−0.427	3.009	16.398
**Eastern China**	PH	216	13.790	1.283	10.241	16.386	−0.319	2.516	5.788
	lnpgdp	216	10.358	0.763	8.445	11.832	−0.285	2.318	7.110
	ML	216	5.877	0.687	4.210	7.400	−0.289	2.728	3.668
	CPI	216	102.124	1.924	97.650	107.780	0.212	2.882	1.749
	EP	216	3.721	1.169	0.358	5.300	−1.031	3.324	39.246
	TP	216	14.308	1.684	9.025	16.970	−0.884	3.646	31.906
	IO	216	9.659	1.218	5.978	11.667	−0.768	3.463	23.158
	SI	216	8.490	1.199	4.644	10.574	−0.691	3.473	19.224
**Central China**	PH	162	13.625	1.309	11.419	15.940	−0.086	1.603	13.354
	lnpgdp	162	9.803	0.706	8.487	11.054	−0.187	1.727	11.873
	ML	162	6.051	0.539	4.740	7.280	0.225	2.431	2.542
	CPI	162	102.262	1.979	98.300	107.190	0.402	2.564	5.644
	EP	162	4.242	0.543	2.810	5.106	−0.052	2.350	2.924
	TP	162	13.554	1.162	10.418	15.762	−0.281	2.611	3.158
	IO	162	9.226	0.869	7.507	11.018	−0.058	2.014	6.659
	SI	162	8.205	0.870	6.367	9.949	−0.096	2.191	4.676
**Western China**	PH	162	13.248	1.410	10.066	15.934	−0.143	2.042	6.751
	lnpgdp	162	9.654	0.771	7.923	11.090	−0.167	1.907	8.822
	ML	162	6.186	0.794	4.260	7.695	−0.801	2.842	17.510
	CPI	162	102.452	2.224	96.700	110.090	0.625	3.583	13.169
	EP	162	3.946	0.667	2.224	4.987	−0.662	2.724	12.359
	TP	162	12.644	1.492	9.371	15.668	−0.047	2.292	3.441
	IO	162	8.606	1.019	6.237	10.675	−0.104	2.390	2.805
	SI	162	7.363	1.161	4.393	9.587	−0.288	2.631	3.151

## Empirical Results

We first test for cross-sectional dependence using Pesaran's cross-section dependence test before moving on to unit root tests (58) (see Table 2). The assumption of cross-sectional independence was used in the early literature on unit root studies (58). However, macrolevel data often deviates from this principle, resulting in low power and size distortions for tests that presume cross-section independence. When studying the correlation between economic growth and public health, the possible cross-sectional dependency is taken into account, as it has been in several studies. Stata 15 was used in this study to perform Levin–Lin–Chu tests to guarantee the validity of test results (LLC). The cross-sectional to time-series ratio to reach zero established a unit root testing that assumes the panel's time-series dimension must expand faster than the cross-sectional dimension. You can check whether each variable has a unit root or only individual intercept using individual intercept and time choices. The unit root test's null hypothesis is that a unit root exists. The stationarity test shows that the variables in the model are stationary and meet the threshold regression modeling criteria, ruling out the unit root hypothesis (58). As a result, we investigate the panel threshold regression model (PTRM).

**Table 2:** Panel unit root tests.

**Variables**	**Panel augmented Dickey–Fuller test**			
	**Levin–Lin–Chu**		**Im–Pesaran–Shin**	
	***T*-statistic**	***P*-value**	***T*-statistic**	***P*-value**
PH	−5.177^***^	0.000	−3.933^***^	0.0001
Lnpgdp	−9.063^***^	0.000	−3.837^***^	0.000
ML	−3.218^***^	0.001	−3.816^***^	0.0001
CPI	−10.477^***^	0.000	−1.955^**^	0.074
EP	−1.772^**^	0.081	−1.988^**^	0.059
TP	−12.272^***^	0.000	−2.102^**^	0.018
IO	−9.722^***^	0.000	−8.293^***^	0.000
SI	−10.462^***^	0.000	−3.234^***^	0.0006

*** and ** *, respectively, indicates significance at the 1 and 5% level*.

The self-sampling test of the economic growth threshold effect on public health is shown in Table 3. In the regression, we set the number of bootstraps as 1,000 times, ignored the 1% variable in the two segments of the threshold variable, and set the regression gird as 400, which reduced the workload of dismantling molecular samples in the model to a certain extent. According to the threshold theory of Hansen (58), in the single-threshold panel model, the F statistic is 54.7, and the corresponding *P-*value is 0.0333. It rejects the original hypothesis of the linear model. That is, there is at least a threshold that is significant at a 5% confidence level. The regression results of the panel double-threshold model showed that the *F-statistic* value for testing the double-threshold effect is 9.5, and the corresponding *P*-value is 0.874. The null hypothesis here is that only one threshold is accepted.

**Table 3:** Tests for threshold effects between GDP and public health.

**Threshold variable**	**Threshold**	**F-stat**	**Prob**	**Crit10**	**Crit5**	**Crit1**
Lnpgdp	Single	54.700^**^	0.039	43.261	51.314	68.675
	Double	9.500	0.874	39.115	45.184	58.596

** *indicates significance at the 5% level*.

According to the regression results of variables in different threshold intervals given in Table 4, when the threshold value is not exceeded, the regression coefficient of economic growth to public health is 1.146. When the threshold value of economic growth is exceeded, the regression coefficient increases to 1.167, as well as the coefficient value before and after the threshold at the 1% significant level. This suggests that China's economic growth has a clear threshold impact on public health and that the larger the elasticity of economic growth on public health after the threshold is reached, the more successful the public health progress would be. No question increasing income levels would help to boost public health. Economic growth leads to increased wages and a higher quality of life, which improves health (36).

**Table 4:** Estimated results of the economic growth threshold model.

**Variable**	**Coef**	**Std. err**.	**t**	***P* > |t|**	**[95% conf. interval]**	
ML	0.077^***^	0.022	3.44	0.001	0.033	0.121
CPI	−0.010^***^	0.004	−2.57	0.001	−0.017	−0.002
EP	−0.087^***^	0.0257	−3.39	0.001	−0.137	−0.036
TP	0.209^***^	0.039	5.36	0.000	0.132	0.285
IO	−0.175^**^	0.095	−1.84	0.066	−0.362	0.0115
SI	0.336^***^	0.064	5.25	0.000	0.210	0.462
PH(lnpgdp ≤ 9.430)	1.146^***^	0.116	9.87	0.000	0.918	1.374
PH(lnpgdp > 9.430)	1.167^***^	0.115	10.08	0.000	0.940	1.395
_cons	−1.645^***^	0.572	−2.87	0.004	−2.769	−0.520

*** and ** *, respectively, indicate significance at the 1 and 5% levels*.

As is shown in Table 1, there are large discrepancies in terms of economic development across different regions (59). Furthermore, the natural resources, economic structure, regional development policies, and systems also vary across economic zones, and the gap between the economic development of the three economic zones is widening in the long term (59). As a result, when analyzing Chinese data, a regional analysis is sufficient.

Table 5 shows the self-sampled impact of economic growth on public health in the eastern, central, and western regions. In the eastern region, the *F-statistic* value of the single threshold effect in the eastern region is 19.15, and the corresponding *P*-value is 0.2767. The hypothesis of the linear model is adopted. The single economic growth threshold for the central region is 9.595, which is important at a significance level of 5%, and the F-statistic is 39.06. The linear model's null hypothesis is rejected. A further test of the double threshold effect showed that the *F-statistic* value was 17.06, and the corresponding *P*-value was 0.130. The null hypothesis there is that only one threshold is accepted. In the regression results of the single-threshold panel model in the western region, the *F-statistic* value is 23.97, and the corresponding *P*-value is 0.080, which also rejects the null hypothesis of the line model and is significant at the confidence level of 10%. The *F-statistic* value of testing the double-threshold effect is 6.07, and the corresponding *P*-value is 0.673, not significant. The null hypothesis here is that only one threshold is accepted.

**Table 5:** Tests for threshold effects between GDP and public health in the subregion.

**Region**	**Threshold**	**F-stat**	**Prob**	**Crit10**	**Crit5**	**Crit1**
Eastern China	Single	19.15	0.2767	28.846	33.783	42.218
Central China	Single	39.06^***^	0.003	24.340	28.557	36.722
	Double	17.06	0.130	17.94	21.582	28.659
Western China	Single	23.97^*^	0.080	22.174	25.290	32.166
	Double	6.07	0.673	18.096	21.857	28.354

*** and * *, respectively, indicate significance at the 1 and 10% levels*.

Since there is no threshold between economic growth and public health in the eastern region, it meets the linear relationship and passes the Hausman test, and the fixed-effect regression is conducted for the eastern. Regression results are shown in Table 6. In the eastern region, economic growth significantly affects public health, and it is significant at the level of 1% with a coefficient of 1.249. With a total area of 1,294,000 km^2^, China's east coast accounts for 13.5% of the country's total area. Fossil fuels, seafood, iron ore, and minerals are abundant in the eastern region (44). Local governments are responsible for most of China's medical and health expenditure, and the eastern region has a high level of economic development and is able to support public resource investment. Eastern governments have a sense of development, making local governments pay more attention to the people's livelihood, and the corresponding medical and health spending has also had increased (48). The eastern region has the largest number of medical practitioners, health personnel, and medical and health institutions among the three regions. It also has the most medical-related professional knowledge, the medical security system is in place, and the people also have health awareness. At the same time, the budget allocations of directly affiliated health institutions and medical research institutions are mainly borne by the central government. Most of these institutions are located in the eastern regions. As a result, the eastern region is better able to meet people's demand for health spending. However, there is no non-linear structural change of threshold characteristics.

**Table 6:** Regression of fixed effect of panel data in Eastern China.

**Region**	**Variable**	**Coef**	**Std. err**.	**t**	***P* > |t|**	**[95% conf. interval]**	
Eastern China	Lnpgdp	1.249^***^	0.120	10.4	0.000	1.013	1.485
	ML	0.091^***^	0.023	3.91	0.000	0.045	0.137
	CPI	−0.008^**^	0.004	−2	0.047	−0.016	0.000
	EP	−0.046^*^	0.026	−1.75	0.080	−0.097	0.006
	TP	0.175^***^	0.040	4.34	0.000	0.096	0.254
	IO	0.339^***^	0.067	5.08	0.000	0.208	0.471
	SI	−0.114	0.099	−1.16	0.248	−0.308	0.080
	_cons	−3.011^***^	0.558	−5.39	0.000	−4.108	−1.914

***, **, and * *, respectively, indicate significance at the 1, 5, and 10% levels*.

Table 7 illustrates the estimation results of the threshold model in the western and central regions, respectively. Among them, the threshold value of economic growth in the central region is 9.595, and the threshold value of economic growth in the western region is 9.448, which is higher in the central region than in the western region. The regression coefficients of economic growth to public health in central and western regions are significant at a 1% level. When the economic growth in the central region exceeds the threshold value, the impact factor increases from 1.428 to 1.463. When the western region's economic growth exceeds the threshold value, the impact factor increases from 0.741 to 0.766. It shows that economic growth contributes more and more to public health in China's central and western regions and has the greatest effect on the central region.

**Table 7:** Estimated results of the threshold model for economic growth in Central and Western China.

**Region**	**Variable**	**Coef**	**Std. err**.	**t**	***P* > |t|**	**[95% conf. interval]**	
Central China	ML	0.107^**^	0.042	2.53	0.012	0.023	0.191
	CPI	−0.024^***^	0.006	−3.49	0.001	−0.037	−0.010
	EP	−0.036	0.054	−0.67	0.504	−0.143	0.070
	TP	0.113	0.085	1.32	0.187	−0.055	0.281
	IO	0.224^**^	0.111	2.01	0.046	0.003	0.444
	SI	−0.248^*^	0.127	−1.96	0.052	−0.499	0.002
	lnpgdp(lnpgdp ≤ 9.595)	1.428^***^	0.192	7.42	0.000	1.047	1.808
	lnpgdp(lnpgdp > 9.595)	1.463^***^	0.191	7.64	0.000	1.085	1.84
	_cons	−0.182	0.928	−0.20	0.844	−2.01	1.652
Western China	ML	0.060	0.042	1.45	0.150	−0.022	0.143
	CPI	−0.001	0.006	−0.29	0.770	−0.014	0.010
	EP	−0.095^*^	0.051	−1.85	0.067	−0.198	0.006
	TP	0.036	0.063	0.57	0.570	−0.088	0.161
	IO	0.544^***^	0.104	5.23	0.000	0.338	0.751
	SI	0.138	0.181	0.77	0.445	−0.219	0.496
	lnpgdp(lnpgdp ≤ 9.448)	0.741^***^	0.191	3.86	0.000	0.361	1.120
	lnpgdp(lnpgdp > 9.448)	0.766^***^	0.191	3.99	0.000	0.386	1.144
	_cons	−0.027	1.140	−0.02	0.981	−2.281	2.227

***, **, and * *, respectively, indicate significance at the 1, 5, and 10% levels*.

The central area is rich in metal and non-metal resources, which has led to the growth of the heavy industry (27). Energy-intensive industries have made substantial progress in central regions since the introduction of the central region rise strategy (27). There are still many shortcomings in economic development, such as serious resource consumption, low output efficiency, excessive pollution discharge, weak capacity for independent innovation, and some overcapacity. The industrial structure in the central region consists of a high proportion of steel, petrochemical, cement, and other industries with high energy load (50). Along with the economic development, people's living standards have been improved to a certain extent, but health expenditure has not been better met. As the economy grows, its contribution to health spending changes structurally.

The terrain in the western area is challenging to navigate, and transportation and investment are scarce. The western region covers a wide area and has lower levels of urbanization, health financial services, and human resources than the eastern and central regions. China implemented the Western Development Strategy (WDS) in the year 2000. In its early years, WDS helped the economic development of western regions and sparked rapid growth. The WDS, for example, has increased the annual growth rate of the western regions by about 1.5% since 2000, according to (59). Besides that, since the WDS reduces the overall corporate income tax rate by 11.5%, it has prompted a rise in labor, human capital, and fixed assets, improving existing companies' competitiveness (23). However, compared with the eastern and central regions, there is still a certain gap. The per capita GDP of the western region is lower than the national average level, which is in line with the objective reality that the western region is an underdeveloped region among the three major regions in the country (22). Regional financial support is insufficient, leading to the western public health services being still relatively backward.

Last but not least, most of the provinces in western China rely on resources to develop their economy, and the industrial chain is not perfect and too short. There were policy traps in the WDS (22) implementation. Local governments have emphasized fixed-asset investments and energy extraction while neglecting structural changes and soft environmental construction, resulting in inadequate healthcare spending in this area. Therefore, when the economy grows in the western region, its promoting effect on health expenditure will also significantly change. However, the development of the western region is far from reaching the development level of the central region, and its promoting effect will also be smaller than that of the central region.

## Conclusions

This paper examines the effect of economic growth on public health in China's three major geographic regions. Economic growth is described in our study as real GDP per capita (GDP). We use a PTRM and a linear regression model to examine the heterogeneity of economic growth on public health in China due to significant regional variations. Because of the high level of economic growth in the eastern region, the development of public health has been normalized, and there is no threshold. The economic growth thresholds in the central and western regions are 9.595 and 9.448, respectively. When economic growth exceeds this threshold, both the effect of economic growth on public health and economic growth on public health will increase instantly. Since the western region's economic development lags behind that of the central region, the promotion impact would be less than that of the central region. This suggests that a partial reallocation of medical services to China's central and western regions makes sense, as it encourages economic growth and thus improves public health. The empirical model described in this paper may be used as a basis for future research into regional heterogeneity in China or other countries.

## Data Availability Statement

The original contributions presented in the study are included in the article/supplementary material, further inquiries can be directed to the corresponding author/s.

## Author Contributions

X-TN: conceptualization, software, data curation, writing—original draft preparation. Y-CY: methodology, visualization, investigation. Y-CW: writing—reviewing and editing. All authors contributed to the article and approved the submitted version.

## Conflict of Interest

The authors declare that the research was conducted in the absence of any commercial or financial relationships that could be construed as a potential conflict of interest.
